# Supplementary material for: The influence of carbon dioxide on cerebral metabolism and oxygen consumption: combining multimodal monitoring with dynamic systems modelling
Source: Biol Open. 2024 Jan 5;13(1):bio060087. doi: 10.1242/bio.060087 (PMC10810564; doi:10.1242/bio.060087)
Supplement: Supplementary information [file biolopen-13-060087-s1.pdf]

1 Overview

Modified BrainSignals model for Highton et al CO2 paper.

- 10 differential state variables
- 3 algebraic state variables
- 48 intermediate variables
- 79 independent parameters
- 60 derived parameters
- 4 declared inputs
- 43 default outputs

2 Differential Equations

$$\frac{\mathrm{d}Cu_{A,o}}{\mathrm{d}t} = 4f_2 - 4f_1 \tag{1}$$

$$\frac{\mathrm{d}a_{3,r}}{\mathrm{d}t} = 4f_2 - 4f_3 \tag{2}$$

$$\frac{\mathrm{d}\psi}{\mathrm{d}t} = \frac{p_2 f_2 + p_1 f_1 + p_3 f_3 - L}{C_{im}} \tag{3}$$

$$\frac{\mathrm{d}H^+}{\mathrm{d}t} = \frac{1}{R_{Hi}} L - \frac{p_3}{R_{Hi}} f_3 - \frac{p_2}{R_{Hi}} f_2 - \frac{p_1}{R_{Hi}} f_1 \tag{4}$$

$$\frac{\mathrm{d}O_2}{\mathrm{d}t} = \frac{1}{Vol_{mit}} J_{O_2} - f_3 \tag{5}$$

$$\frac{\mathrm{d}v_{CO_2}}{\mathrm{d}t} = \frac{1}{\tau_{CO_2}} \left(Pa_{CO_2} - v_{CO_2}\right) \tag{6}$$

Biology Open • Supplementary information

$$\frac{\mathrm{d}\nu_{CO_2,2}}{\mathrm{d}t} = \frac{1}{\tau_{CO_2,2}} \left(Pa_{CO_2} - \nu_{CO_2,2}\right) \tag{7}$$

$$\frac{\mathrm{d}\nu_{O_2}}{\mathrm{d}t} = \frac{1}{\tau_{O_2}} \left(O_{2,c} - \nu_{O_2}\right) \tag{8}$$

$$\frac{\mathrm{d}\nu_{P_a}}{\mathrm{d}t} = \frac{1}{\tau_{P_a}} \left(P_a - \nu_{P_a}\right) \tag{9}$$

$$\frac{\mathrm{d}\nu_u}{\mathrm{d}t} = \frac{1}{\tau_u} \left(u - \nu_u\right) \tag{10}$$

3 Algebraic Equations

$$\phi \left(\frac{S_{c,O_2}}{1 - S_{c,O_2}}\right)^{\frac{1}{n_h}} - O_{2,c} = 0 \tag{11}$$

$$T_e + T_m - \left(P_1 - P_{ic}\right) r = 0 \tag{12}$$

$$CBF \left(HbO_{2,a} - HbO_{2,v}\right) - J_{O_2} = 0 \tag{13}$$

4 Chemical Reactions

$$\xrightarrow{L} \frac{1}{R_{Hi}} \mathrm{H}^+ \tag{14}$$

$$\xrightarrow{J_{O_2}} \frac{1}{Vol_{mit}} \mathrm{O}_2 \tag{15}$$

$$\frac{p_2}{R_{Hi}} \mathrm{H}^+ \xrightarrow{f_2} 4\,\mathrm{Cu}_{\mathrm{A,o}} + 4\,\mathrm{a}_{\mathrm{3,r}} \tag{16}$$

$$4\,\mathrm{Cu}_{\mathrm{A,o}} + \frac{p_1}{R_{Hi}} \mathrm{H}^+ \xrightarrow{f_1} \tag{17}$$

$$\mathrm{O}_2 + 4\,\mathrm{a}_{\mathrm{3,r}} + \frac{p_3}{R_{Hi}} \mathrm{H}^+ \xrightarrow{f_3} \tag{18}$$

Biology Open • Supplementary information

5 State Variables

$Cu_{A,o}$

Implementation Name: `a`
Units: mM
Initial value: $Cu_{A,o,n}$
Concentration of oxidised cytochrome c oxidase.

$a_{3,r}$

Implementation Name: `bred`
Units: mM
Initial value: $a_{3,r,n}$
Concentration of reduced cytochrome a_3.

ψ

Implementation Name: `Dpsi`
Units: mV
Initial value: ψ_n
Mitochondrial inner membrane potential. Varies as charge (in the form of protons) is transferred across the membrane capacitance.

H^+

Implementation Name: `H`
Units: mM
Initial value: H_n^+
Mitochondrial proton concentration.

O_2

Implementation Name: `O2`
Units: mM
Initial value: $O_{2,n}$
Mitochondrial oxygen concentration.

$O_{2,c}$

Implementation Name: `O2c`
Units: mM
Initial value: $O_{2,c,n}$
Capillary oxygen concentration.

r

Implementation Name: `r`
Units: cm
Initial value: r_n
Typical blood vessel radius.

v_{CO_2}

Implementation Name: `v_c`
Units: mmHg
Initial value: $v_{CO_2,n}$
Filtered CO_2 partial pressure.

$v_{CO_2,2}$

Implementation Name: `v_c2`
Units: mmHg
Initial value: $v_{CO_2,2,n}$
Filtered CO_2 partial pressure affecting metabolism.

v_{O_2}

Implementation Name: `v_o`
Units: mM

Biology Open • Supplementary information

Initial value: $\nu_{O_2,n}$
Filtered capillary oxygen concentration.

ν_{P_a}
Implementation Name: `v_p`
Units: mmHg
Initial value: $\nu_{P_a,n}$
Filtered arterial blood pressure.

ν_u
Implementation Name: `v_u`
Units: dimensionless
Initial value: $\nu_{u,n}$
Filtered demand.

$HbO_{2,v}$
Implementation Name: `XOv`
Units: mM
Initial value: $HbO_{2,v,n}$
Venous concentration of oxygen bound to haemoglobin.

6 Intermediate Variables

$Cu_{A,r} = CCO_{tot} - Cu_{A,o}$
Implementation Name: `ared`
Units: mM
Initial value: 0
Concentration of reduced Cu_A.

$a_{3,o} = CCO_{tot} - a_{3,r}$
Implementation Name: `b`
Units: mM
Initial value: 0
Concentration of oxidised cytochrome a_3.

$$C_{0,i} = \frac{10^{-pH_m} - 10^{-pH_m - dpH}}{dpH}$$
Implementation Name: `C_0i`
Units: dimensionless
Initial value: 0
Natural buffering capacity of protons in mitochondria.

$$C_{NADH} = \frac{Z}{2}\log 10 \left(\frac{1}{\frac{NAD}{NADH}} \right)$$
Implementation Name: `C_NADH`
Units: mV
Initial value: 0
Excess redox potential for NADH at normal demand.

$CBF = G\,(P_a - P_v)$
Implementation Name: `CBF`
Units: $ml_{blood}\ ml_{brain}^{-1}\ s^{-1}$
Initial value: CBF_n
Cerebral blood flow.

$\Delta oxCCO = \Delta oxCCO_{off} + 1000 Vol_{mit}\,(Cu_{A,o} - Cu_{A,o,n})$
Implementation Name: `CCO`

Biology Open • Supplementary information

Units: uM
Initial value: 0
Cytochrome c oxidase signal measured by NIRS.

$CMRO_2 = f_3 \, Vol_{mit}$
Implementation Name: `CMRO2`
Units: mM s^{-1}
Initial value: 0
Rate of cerebral oxygen metabolism.

$\Delta p = \psi + Z \, (pH_m - pH_o)$
Implementation Name: `Dp`
Units: mV
Initial value: 0
Proton motive force across the mitochondrial inner membrane.

$E_{1,NADH} = \mathcal{E}_0(\mathrm{Cu}_A) - \mathcal{E}_0(\mathrm{NADH}) + C_{NADH}$
Implementation Name: `E1NADH`
Units: mV
Initial value: 0
Value of E_1 when the reducing substrate is NADH.

$E_1 = E_{1,NADH}$
Implementation Name: `E_1`
Units: mV
Initial value: 0
The energy provided by electron transfer to $\mathrm{Cu}_{A,r}$.

$\eta = R_{P_a} \left(\frac{v_{P_a}}{v_{P_a,n}} - 1 \right) + R_{O_2} \left(\frac{v_{O_2}}{v_{O_2,n}} - 1 \right) + R_{CO_2} \left(1 - \frac{v_{CO_2}}{v_{CO_2,n}} \right) + R_u \left(1 - \frac{v_u}{v_{u,n}} \right)$
Implementation Name: `eta`
Units: dimensionless
Initial value: 0
Merged autoregulation stimulus.

$f_1 = k_1 \, Cu_{A,o} - k_{-1} \, Cu_{A,r}$
Implementation Name: `f1`
Units: mM s^{-1}
Initial value: 0
Reaction rate for the reduction of Cu_A.

$f_2 = k_2 \, Cu_{A,r} \, a_{3,o} - k_{-2} \, Cu_{A,o} \, a_{3,r}$
Implementation Name: `f2`
Units: mM s^{-1}
Initial value: 0
Reaction rate for the reduction of a_3.

$f_3 = \frac{k_3 \, O_2 \, a_{3,r} \exp\left(-c_3 \, (\Delta p - \Delta p_{30})\right)}{1 + \exp\left(-c_3 \, (\Delta p - \Delta p_{30})\right)}$
Implementation Name: `f3`
Units: mM s^{-1}
Initial value: 0
Reaction rate for the reduction of O_2.

$G = K_G \, r^4$
Implementation Name: `G`
Units: $\mathrm{ml}_{blood} \, \mathrm{ml}_{brain}^{-1} \, \mathrm{mmHg}^{-1} \, \mathrm{s}^{-1}$
Initial value: 0
Effective conductance of the whole blood flow compartment.

Biology Open • Supplementary information

$h = \text{sqrt}\left(r^2 + 2r_0\, h_0 + h_0^2\right) - r$
Implementation Name: `h`
Units: cm
Initial value: h_n
Thickness of the blood vessel walls.

$HbO_2 = \left(V_a\, HbO_{2,a} + V_v\, HbO_{2,v}\right)\, blood_{hb}$
Implementation Name: `HbO2`
Units: uM
Initial value: 0
Oxygenated haemoglobin signal measured by NIRS.

$HbT = \left(V_a + V_v\right)\, Hb_{tot}\, blood_{hb}$
Implementation Name: `HbT`
Units: uM
Initial value: 0
Total haemoglobin signal measured by NIRS.

$HHb = HbT - HbO_2$
Implementation Name: `HHb`
Units: uM
Initial value: 0
Deoxygenated haemoglobin signal measured by NIRS.

$J_{O_2} = \text{fmin}\left(D_{O_2}\,\left(O_{2,c} - O_2\right), CBF\, HbO_{2,a}\right)$
Implementation Name: `J_O2`
Units: mM s^{-1}
Initial value: 0
Oxygen flux from blood to tissue.

$k_1 = k_{1,0}\exp\left(-c_{k_1}\,\left(\Delta p - \Delta p_n\right)\right)$
Implementation Name: `k1`
Units: s^{-1}
Initial value: 0
Forward reaction rate for the reduction of Cu_A.

$k_{1,0} = \frac{k_{1,n}\, NADH}{NADH_n}$
Implementation Name: `k10`
Units: s^{-1}
Initial value: 0
Forward reaction rate for the reduction of Cu_A at normal Δp.

$k_2 = k_{2,n}\exp\left(-c_{k_2}\,\left(\Delta p - \Delta p_n\right)\right)$
Implementation Name: `k2`
Units: s^{-1}
Initial value: 0
Forward reaction rate for the reduction of a_3.

$K_{eq_1} = 10^{\frac{-1}{Z}\left(\frac{p_1 \Delta p}{4} - E_1\right)}$
Implementation Name: `Keq1`
Units: dimensionless
Initial value: 0
Equilibrium constant for the Cu_A reduction reaction.

$K_{eq_2} = 10^{\frac{-1}{Z}\left(\frac{p_2 \Delta p}{4} - E_2\right)}$
Implementation Name: `Keq2`
Units: dimensionless

Biology Open • Supplementary information

Initial value: 0
Equilibrium constant for the a_3 reduction reaction.

$$k_{-1} = \frac{k_1}{K_{eq_1}}$$
Implementation Name: `kn1`
Units: s^{-1}
Initial value: 0
Reverse reaction rate for the reduction of Cu_A.

$$k_{-2} = \frac{k_2}{K_{eq_2}}$$
Implementation Name: `kn2`
Units: s^{-1}
Initial value: 0
Reverse reaction rate for the reduction of a_3.

$$L = L_{CV} + L_{lk}$$
Implementation Name: `L`
Units: mM s^{-1}
Initial value: 0
Rate of proton return to the mitochondrial matrix.

$$L_{CV} = \frac{CV_{inh}\, L_{CV,max}\, (1 - \exp(-\theta))}{1 + r_{CV} \exp(-\theta)}$$
Implementation Name: `L_CV`
Units: mM s^{-1}
Initial value: 0
Rate at which protons re-enter the mitochondrial matrix due to ADP phosphorylation.

$$L_{lk} = k_{unc}\, L_{lk0}\, (\exp(\Delta p\, k_{lk2}) - 1)$$
Implementation Name: `L_lk`
Units: mM s^{-1}
Initial value: 0
Rate at which protons re-enter the mitochondrial matrix via leak channels.

$$\mu = \frac{\mu_{min} + \mu_{max} \exp(\eta)}{1 + \exp(\eta)}$$
Implementation Name: `mu`
Units: dimensionless
Initial value: 0
Effective strength of the autoregulation reponse.

$$NADH = \frac{NAD_{pool}}{1 + \frac{NAD}{NADH}}$$
Implementation Name: `NADH`
Units: mM
Initial value: 0
Concentration of NADH in the mitochondria.

$$\frac{NAD}{NADH} = \frac{supp\, \frac{NAD_n}{NADH_n}}{u^{2D_{NADH}}}$$
Implementation Name: `NADNADHrat`
Units: dimensionless
Initial value: 0
NAD/NADH ratio.

$$pH_m = -\log 10 \left(\frac{H^+}{1000} \right)$$
Implementation Name: `pH_m`

Biology Open • Supplementary information

Units: dimensionless
Initial value: 0
Mitochondrial pH.

$$r_{buffi} = \frac{C_{buffi}}{C_{0,i}}$$

Implementation Name: `r_buffi`
Units: dimensionless
Initial value: 0
Buffering capacity for protons in mitochondria.

$$R_{Hi} = r_{buffi}$$

Implementation Name: `R_Hi`
Units: dimensionless
Initial value: 0
Relative mitochondrial volume for protons, taking into account buffering effect of pH.

$$S_{c,O_2} = \frac{S_{a,O_2} + S_{v,O_2}}{2}$$

Implementation Name: `ScO2`
Units: dimensionless
Initial value: $S_{c,O_2,n}$
Capillary oxygen saturation.

$$\sigma_e = \sigma_{e,0} \left(\exp\left(\frac{K_\sigma \, (r - r_0)}{r_0} \right) - 1 \right) - \sigma_{coll}$$

Implementation Name: `sigma_e`
Units: mm Hg
Initial value: 0
Elastic stress in blood vessel walls.

$$supp = supp_n + supp_{CO_2} \left(v_{CO_2,2} - v_{CO_2,2,n} \right)$$

Implementation Name: `supp`
Units: dimensionless
Initial value: 0
Relative supply of reducing substrate.

$$S_{v,O_2} = \frac{HbO_{2,v}}{Hb_{tot}}$$

Implementation Name: `SvO2`
Units: dimensionless
Initial value: $S_{v,O_2,n}$
Venous oxygen saturation.

$$T_e = \sigma_e \, h$$

Implementation Name: `T_e`
Units: mm Hg cm
Initial value: 0
Elastic tension in the blood vessel walls.

$$T_m = T_{max} \exp\left(- \left| \frac{r - r_m}{r_t - r_m} \right|^{n_m} \right)$$

Implementation Name: `T_m`
Units: mm Hg cm
Initial value: 0
Muscular tension in the blood vessel walls.

$$T_{max} = T_{max,0} \, (1 + k_{aut} \, \mu)$$

Implementation Name: `T_max`
Units: mm Hg cm

Biology Open • Supplementary information

Initial value: 0
Maximal muscular tension in the blood vessel walls.

$\theta = k_{CV} \left(\Delta p + Z \log 10 \left(\nu_u\right) - \Delta p_{CV,0}\right)$
Implementation Name: `theta`
Units: dimensionless
Initial value: 0
Driving force Complex V.

$TOI = \frac{100 HbO_2}{HbT}$
Implementation Name: `TOI`
Units: dimensionless
Initial value: 0
Total oxygenation index.

$u = u_n + \left(\nu_{CO_2,2} - \nu_{CO_2,2,n}\right) u_f$
Implementation Name: `u`
Units: dimensionless
Initial value: 0
Variable indicating relative metabolic demand.

$V_{mca} = CBF\, CBFscale$
Implementation Name: `Vmca`
Units: cm s^{-1}
Initial value: 0
Blood velocity in the middle cerebral artery.

$V_a = V_{a,n} \left(\frac{r}{r_n}\right)^2$
Implementation Name: `Vol_art`
Units: dimensionless
Initial value: 0
Relative arterial blood volume.

7 Parameters

$Cu_{a,frac,n}$
Implementation Name: `a_frac_n`
Units: dimensionless
Initial value: 0.8
Normal oxidised fraction of Cu_A.

$blood_{hb}$
Implementation Name: `blood_hb`
Units: dimensionless
Initial value: 10.00
Factor to convert model haemoglobin concentration to instrumental units. Scales for blood fraction of brain volume, mM to μM, and number of binding sites.

c_3
Implementation Name: `c3`
Units: mV^{-1}
Initial value: 0.11
Parameter controlling the sensitivity of the reduction of a_3 to Δp.

C_{buffi}
Implementation Name: `C_buffi`

Biology Open • Supplementary information

Units: dimensionless
Initial value: 0.022
Buffering capacity of protons in mitochondria.

C_{im}
Implementation Name: C_im
Units: mM mV^{-1}
Initial value: 0.00675
Capacitance of the mitochondrial inner membrane.

CBF_n
Implementation Name: CBFn
Units: ml_{blood} ml_{brain}^{-1} s^{-1}
Initial value: 0.0125
Normal cerebral blood flow.

$CBFscale$
Implementation Name: CBFscale
Units: cm
Initial value: 5000
Scale constant relating blood flow to arterial velocity.

$\Delta oxCCO_{off}$
Implementation Name: CCO_offset
Units: uM
Initial value: 0
Signal offset for the NIRS CCO measurement.

c_{k_1}
Implementation Name: ck1
Units: mV^{-1}
Initial value: 0.01
Parameter controlling sensitivity of k_1 to Δp.

c_{k_2}
Implementation Name: ck2
Units: mV^{-1}
Initial value: 0.02
Parameter controlling sensitivity of k_2 to Δp.

$CMRO_{2,n}$
Implementation Name: CMRO2_n
Units: mM s^{-1}
Initial value: 0.034
Normal metabolic rate of oxygen consumption.

CV_{inh}
Implementation Name: CVinh
Units: dimensionless
Initial value: 1
Control parameter representing the action of Complex V inhibitors.

CCO_{tis}
Implementation Name: cytox_tot_tis
Units: mM
Initial value: 0.0055
Concentration of cytochrome c oxidase in tissue.

D_{NADH}
Implementation Name: D_NADH
Units: dimensionless

Biology Open • Supplementary information

Initial value: 0.01
Scale parameter for the dependence of NADH redox potential on demand.

$D_{O_2,f}$
Implementation Name: D_O2f
Units: dimensionless
Initial value: 1
Scaling factor for O_2 diffusion rate in tissue.

$\Delta p_{3,corr}$
Implementation Name: Dp3_corr
Units: mV
Initial value: -25
Difference between Δp_{30} and normal Δp.

$\Delta p_{CV,0}$
Implementation Name: Dp_CV0
Units: mV
Initial value: 90
Value of Δp at which L_{CV} is zero under normal demand.

dpH
Implementation Name: dpH
Units: dimensionless
Initial value: 0.001
Parameter in the mitochondrial proton buffering relationship.

ψ_n
Implementation Name: Dpsi_n
Units: mV
Initial value: 145
Normal mitochondrial inner membrane potential.

$\mathcal{E}_0(\mathrm{a}_3)$
Implementation Name: E_a30
Units: mV
Initial value: 350
Standard redox potential for cytochrome a_3.

$\mathcal{E}_0(\mathrm{Cu}_A)$
Implementation Name: E_c0
Units: mV
Initial value: 247
Standard redox potential for Cu_A.

$\mathcal{E}_0(\mathrm{NADH})$
Implementation Name: E_N0
Units: mV
Initial value: -320
Standard redox potential for NADH.

h_0
Implementation Name: h_0
Units: cm
Initial value: 0.003
Thickness of the blood vessel walls at which radius is r_0.

$k_{3,0}$
Implementation Name: k30
Units: s^{-1}

Biology Open • Supplementary information

Initial value: $2.5E+5$
Apparent second order rate constant for reduction of O_2 at zero Δp.

k_{aut}
Implementation Name: k_aut
Units: dimensionless
Initial value: 1
Overall functioning of autoregulatory response.

k_{lk2}
Implementation Name: k_lk2
Units: mV^{-1}
Initial value: 0.038
Constant controlling the depending of the leak rate L_{lk} on Δp.

K_σ
Implementation Name: K_sigma
Units: dimensionless
Initial value: 10
Parameter controlling the sensitivity of σ_e to vessel radius.

k_{unc}
Implementation Name: k_unc
Units: dimensionless
Initial value: 1
Control parameter simulating the effect of adding uncouplers to the system.

$L_{CV,0}$
Implementation Name: L_CV0
Units: dimensionless
Initial value: 0.4
Normal Complex V flux as a fraction of maximum possible flux.

$L_{lk,frac}$
Implementation Name: L_lkfrac
Units: dimensionless
Initial value: 0.25
Normal fraction of proton entry into mitochondria which is via leak channels.

μ_{max}
Implementation Name: mu_max
Units: dimensionless
Initial value: 1
Upper bound for the transformed stimulus μ.

μ_{min}
Implementation Name: mu_min
Units: dimensionless
Initial value: -1
Lower bound for the transformed stimulus μ.

μ_n
Implementation Name: mu_n
Units: dimensionless
Initial value: 0
Normal value for the transformed stimulus μ.

n_h
Implementation Name: n_h
Units: dimensionless

Biology Open • Supplementary information

Initial value: 2.5
Hill coefficient for oxygen dissociation from haemoglobin.

n_m
Implementation Name: n_m
Units: dimensionless
Initial value: 1.83
Exponent in the muscular tension relationship.

$\frac{NAD_n}{NADH_n}$
Implementation Name: NADNADHratn
Units: dimensionless
Initial value: 9
Normal NAD/NADH ratio.

NAD_{pool}
Implementation Name: NADpool
Units: dimensionless
Initial value: 3
Relative size of the NAD pool, used to estimate normal mitochondrial NADH.

$O_{2,n}$
Implementation Name: O2_n
Units: mM
Initial value: 0.024
Normal mitochondrial oxygen concentration.

p_2
Implementation Name: p2
Units: dimensionless
Initial value: 4
Proton cost of the reaction reducing a_3.

p_{23}
Implementation Name: p23
Units: dimensionless
Initial value: 8
Total protons removed from the mitochondrial matrix by the reductions of a_3 and O_2.

$P_{a,n}$
Implementation Name: P_an
Units: mmHg
Initial value: 100
Normal arterial blood pressure.

p_{C1}
Implementation Name: p_C1
Units: dimensionless
Initial value: 8
Protons pumped by Complex I.

p_{C3}
Implementation Name: p_C3
Units: dimensionless
Initial value: 4
Protons pumped by Complex III.

P_{ic}
Implementation Name: P_ic
Units: mm Hg

Biology Open • Supplementary information

Initial value: 9.5
Intracranial pressure.

P_{icn}
Implementation Name: `P_icn`
Units: mm Hg
Initial value: 9.5
Normal intracranial pressure.

$P_{v,n}$
Implementation Name: `P_vn`
Units: mmHg
Initial value: 4
Normal venous blood pressure.

$Pa_{CO_{2,n}}$
Implementation Name: `Pa_CO2n`
Units: mmHg
Initial value: 40
Normal arterial partial pressure of carbon dioxide.

$pH_{m,n}$
Implementation Name: `pH_mn`
Units: dimensionless
Initial value: 7.4
Normal mitochondrial pH.

pH_o
Implementation Name: `pH_o`
Units: dimensionless
Initial value: 7
Extra-mitochondrial pH.

$pH_{o,n}$
Implementation Name: `pH_on`
Units: dimensionless
Initial value: 7
Normal extra-mitochondrial pH.

ϕ
Implementation Name: `phi`
Units: mM
Initial value: 0.036
Oxygen concentration at half-maximal saturation.

r_0
Implementation Name: `r_0`
Units: cm
Initial value: 0.0126
Radius in the elastic tension relationship.

R_{CO_2}
Implementation Name: `R_autc`
Units: dimensionless
Initial value: 2.2
Autoregulatory reactivity to carbon dioxide.

R_{O_2}
Implementation Name: `R_auto`
Units: dimensionless

Biology Open • Supplementary information

Initial value: 1.5
Autoregulatory reactivity to oxygen.

R_{P_a}
Implementation Name: R_autp
Units: dimensionless
Initial value: 4
Autoregulatory reactivity to blood pressure.

R_u
Implementation Name: R_autu
Units: dimensionless
Initial value: 0.5
Autoregulatory reactivity to demand.

r_{CV}
Implementation Name: r_CV
Units: dimensionless
Initial value: 5
Parameter controlling the ratio of maximal to minimal rates of oxidative phosphorylation.

r_m
Implementation Name: r_m
Units: cm
Initial value: 0.027
Vessel radius at which muscular tension is maximal.

r_n
Implementation Name: r_n
Units: cm
Initial value: 0.0187
Normal effective blood vessel radius.

r_t
Implementation Name: r_t
Units: cm
Initial value: 0.018
Radius in the muscular tension relationship.

$S_{a,O_2,n}$
Implementation Name: SaO2_n
Units: dimensionless
Initial value: 0.96
Normal arterial oxygen saturation.

σ_{coll}
Implementation Name: sigma_coll
Units: mm Hg
Initial value: 62.79
Pressure at which blood vessels collapse.

$\sigma_{e,0}$
Implementation Name: sigma_e0
Units: mm Hg
Initial value: 0.1425
Parameter in the elastic tension relationship.

$supp_n$
Implementation Name: supp_n
Units: dimensionless

Biology Open • Supplementary information

Initial value: 1
Normal relative supply of reducing substrate.

$supp_{CO_2}$
Implementation Name: `supps`
Units: dimensionless
Initial value: 0
Dependence of metabolic CO_2 on supply of reducing substrate.

t
Implementation Name: `t`
Units: s
Initial value: 0
Time over which the system evolves.

τ_{CO_2}
Implementation Name: `t_c`
Units: s
Initial value: 5
Filter time constant for autoregulatory effect of carbon dioxide.

$\tau_{CO_2,2}$
Implementation Name: `t_c2`
Units: s
Initial value: 5
Filter time constant for effect of carbon dioxide on metabolism.

τ_{O_2}
Implementation Name: `t_o`
Units: s
Initial value: 20
Filter time constant for stimulus effect of capillary oxygen.

τ_{P_a}
Implementation Name: `t_p`
Units: s
Initial value: 5
Filter time constant for stimulus effect of blood pressure.

τ_u
Implementation Name: `t_u`
Units: s
Initial value: 0.5
Filter time constant for stimulus effect of demand.

u_n
Implementation Name: `u_n`
Units: dimensionless
Initial value: 1
Normal demand.

u_f
Implementation Name: `uf`
Units: dimensionless
Initial value: 0
Parameter specifying influence of CO_2 on metabolic demand.

$VArat_n$
Implementation Name: `VArat_n`
Units: dimensionless

Biology Open • Supplementary information

Initial value: 3
Normal volume ratio of veins to arteries in brain tissue.

Vol_{mit}
Implementation Name: Vol_mit
Units: dimensionless
Initial value: 0.067
Fraction of brain tissue volume that is mitochondria.

$V_{tot,n}$
Implementation Name: Vol_totn
Units: dimensionless
Initial value: 1
Normal total blood volume.

Hb_{tot}
Implementation Name: Xtot
Units: mM
Initial value: 9.1
Total concentration of haemoglobin O_2 binding sites in blood (4 times haemoglobin concentration).

$Hb_{tot,n}$
Implementation Name: Xtot_n
Units: mM
Initial value: 9.1
Normal total concentration of haemoglobin O_2 binding sites in blood (4 times haemoglobin concentration).

Z
Implementation Name: Z
Units: mV
Initial value: 59.028
Proportionality constant in calculation of driving forces due to concentration differences. Defined as RT/F, where F is Faraday's constant, R the ideal gas constant and T the absolute temperature.

8 Derived Parameters

$Cu_{A,o,n} = CCO_{tot}\, Cu_{a,frac,n}$
Implementation Name: a_n
Units: mM
Initial value: 0
Normal concentration of oxidised cytochrome c oxidase.

$Cu_{A,r,n} = CCO_{tot} - Cu_{A,o,n}$
Implementation Name: ared_n
Units: mM
Initial value: 0
Normal concentration of reduced Cu_A.

$a_{3,o,n} = CCO_{tot} - a_{3,r,n}$
Implementation Name: b_n
Units: mM
Initial value: 0
Normal concentration of oxidised cytochrome a_3.

Biology Open • Supplementary information

$a_{3,r,n}$
Implementation Name: `bred_n`
Units: mM
Initial value: $\frac{\frac{\frac{f_n}{k_3}}{O_{2,n}}}{\frac{\exp(-c_3\,(\Delta p_n - \Delta p_{30}))}{1+\exp(-c_3\,(\Delta p_n - \Delta p_{30}))}}$
Normal concentration of reduced cytochrome a3.

$C_{NADH,n}$
Implementation Name: `C_NADH_n`
Units: mV
Initial value: $\frac{Z}{2}\,\mathrm{log10}\,\left(\frac{1}{\frac{NAD_n}{NADH_n}}\right)$
Normal value of C_{NADH}.

$CCO_{tot} = \frac{CCO_{tis}}{Vol_{mit}}$
Implementation Name: `cytox_tot`
Units: mM
Initial value: 0
Concentration of cytochrome c oxidase in mitochondria.

$D_{O_2} = \frac{J_{O_{2,n}}\,D_{O_{2,f}}}{O_{2,c,n} - O_{2,n}}$
Implementation Name: `D_O2`
Units: s^{-1}
Initial value: 0
Diffusion rate for oxygen between capillaries and mitochondria.

$\Delta p_{30} = \Delta p_n + \Delta p_{3,corr}$
Implementation Name: `Dp_30`
Units: mV
Initial value: 0
Value of Δp to which a_3 reduction reaction is maximally sensitive.

Δp_n
Implementation Name: `Dp_n`
Units: mV
Initial value: $\psi_n + Z\,\Delta pH_n$
Normal value of Δp

ΔpH_n
Implementation Name: `DpH_n`
Units: dimensionless
Initial value: $pH_{m,n} - pH_{o,n}$
Normal pH difference across the mitochondrial inner membrane.

$E_{1,NADH,n}$
Implementation Name: `E1NADH_n`
Units: mV
Initial value: $\mathcal{E}_0(\mathrm{Cu}_A) - \mathcal{E}_0(\mathrm{NADH}) + C_{NADH,n}$
Normal value of $E_{1,NADH}$.

$E_{1,n}$
Implementation Name: `E_1n`
Units: mV
Initial value: $E_{1,NADH,n}$
Normal value of E_1.

E_2

Biology Open • Supplementary information

Implementation Name: `E_2`
Units: mV
Initial value: $\mathcal{E}_0(a_3) - \mathcal{E}_0(Cu_A)$
Energy provided by the transfer of four electrons from $Cu_{A,r}$ to $a_{3,o}$.

f_n

Implementation Name: `f_n`
Units: mM s^{-1}
Initial value: $\frac{CMRO_{2,n}}{Vol_{mit}}$
Normal resting value of f_1 and f_2.

$G_n = \frac{CBF_n}{P_{a,n} - P_{v,n}}$

Implementation Name: `Gn`
Units: $ml_{blood}\ ml_{brain}^{-1}\ mmHg^{-1}\ s^{-1}$
Initial value: 0
Normal blood vessel conductance.

H_n^+

Implementation Name: `H_n`
Units: mM
Initial value: $10^{3-pH_{m,n}}$
Normal mitochondrial proton concentration.

h_n

Implementation Name: `h_n`
Units: cm
Initial value: $\mathrm{sqrt}\left(r_n^2 + 2r_0\, h_0 + h_0^2\right) - r_n$
Normal thickness of the blood vessel walls.

$J_{O_{2,n}}$

Implementation Name: `J_O2n`
Units: mM s^{-1}
Initial value: $CMRO_{2,n}$
Normal oxygen flux from blood to tissue.

$k_{1,n}$

Implementation Name: `k1_n`
Units: s^{-1}
Initial value: $\frac{f_n}{Cu_{A,o,n} - \frac{1}{K_{eq_1,n}} Cu_{A,r,n}}$
Forward reaction rate for the reduction of Cu_A at normal Δp and NADH.

$k_{2,n}$

Implementation Name: `k2_n`
Units: s^{-1}
Initial value: $\frac{f_n}{Cu_{A,r,n}\, a_{3,o,n} - \frac{1}{K_{eq_2,n}} Cu_{A,o,n}\, a_{3,r,n}}$
Normal forward reaction rate for the reduction of a_3.

$k_3 = \frac{k_{3,0}}{\frac{\exp(-c_3 - \Delta p_{30})}{1+\exp(-c_3 - \Delta p_{30})}}$

Implementation Name: `k3`
Units: s^{-1}
Initial value: 0
Forward reaction rate for the reduction of O_2.

$K_G = \frac{G_n}{r_n^4}$

Biology Open • Supplementary information

Implementation Name: K_G
Units: $\text{ml}_{blood}\ \text{ml}_{brain}^{-1}\ \text{mmHg}^{-1}\ \text{s}^{-1}\ \text{cm}^{-4}$
Initial value: 0
Proportionality constant in Poiseuille relation for conductance.

$$k_{CV} = \frac{-1}{\Delta p_n - \Delta p_{CV,0}} \log\left(\frac{1 - L_{CV,0}}{1 + r_{CV}\, L_{CV,0}}\right)$$

Implementation Name: kCV
Units: mV^{-1}
Initial value: 0
Parameter controlling the sensitivity of Complex V flux to driving force.

$K_{eq_1,n}$
Implementation Name: Keq1_n
Units: dimensionless
Initial value: $10^{\frac{-1}{Z}\left(\frac{p_1 \Delta p_n}{4} - E_{1,n}\right)}$
Normal value of the equilibrium constant for the Cu_A reduction reaction.

$K_{eq_2,n}$
Implementation Name: Keq2_n
Units: dimensionless
Initial value: $10^{\frac{-1}{Z}\left(\frac{p_2 \Delta p_n}{4} - E_2\right)}$
Normal value of the equilibrium constant for the a_3 reduction reaction.

$L_{CV,frac} = 1 - L_{lk,frac}$
Implementation Name: L_CVfrac
Units: dimensionless
Initial value: 0
Normal fraction of proton entry into mitochondria which is due to ADP phosphorylation.

$$L_{CV,max} = \frac{L_{CV,n}}{L_{CV,0}}$$

Implementation Name: L_CVmax
Units: mM s^{-1}
Initial value: 0
The maximum rate of proton flow through Complex V.

$L_{CV,n}$
Implementation Name: L_CVn
Units: mM s^{-1}
Initial value: $L_n\, L_{CV,frac}$
The resting flow of protons into the matrix through Complex V.

L_{lk0}
Implementation Name: L_lk0
Units: mM s^{-1}
Initial value: $\dfrac{L_{lk,n}}{\exp\left(\Delta p_n\, k_{lk2}\right) - 1}$
Constant controlling the depending of the leak rate L_{lk} on Δp.

$L_{lk,n}$
Implementation Name: L_lkn
Units: mM s^{-1}
Initial value: $L_n\, L_{lk,frac}$
The resting flow of protons into the matrix via leak channels.

$L_n = p_{tot}\, f_n$
Implementation Name: L_n
Units: mM s^{-1}

Biology Open • Supplementary information

Initial value: 0
The normal total flow of protons back into mitochondria.

$NADH_n$
Implementation Name: `NADHn`
Units: mM
Initial value: $\frac{NAD_{pool}}{1+\frac{NAD_n}{NADH_n}}$
Normal concentration of NADH in the mitochondria.

$O_{2,c,n}$
Implementation Name: `O2c_n`
Units: mM
Initial value: $\phi\left(\frac{S_{c,O_2,n}}{1-S_{c,O_2,n}}\right)^{\frac{1}{n_h}}$
Normal capillary oxygen concentration.

$p_1 = p_{tot} - p_{23}$
Implementation Name: `p1`
Units: dimensionless
Initial value: 0
Proton cost of the reaction reducing Cu_A.

p_3
Implementation Name: `p3`
Units: dimensionless
Initial value: $p_{23} - p_2$
Proton cost of the reaction reducing O_2.

$P_1 = \frac{P_a + P_v}{2}$
Implementation Name: `P_1`
Units: mm Hg
Initial value: $P_{1,n}$
Average pressure in the blood vessels.

$P_{1,n} = \frac{P_{a,n} + P_{v,n}}{2}$
Implementation Name: `P_1n`
Units: mm Hg
Initial value: 0
Normal value for the average pressure in the blood vessels.

P_a
Implementation Name: `P_a`
Units: mmHg
Initial value: $P_{a,n}$
Mean arterial blood pressure.

p_{tot}
Implementation Name: `p_tot`
Units: dimensionless
Initial value: $p_{tot,NADH}$
Total protons removed from the mitochondrial matrix by the three modelled electron transport reactions.

$p_{tot,NADH} = p_{C1} + p_{C3} + p_{23}$
Implementation Name: `p_totNADH`
Units: dimensionless
Initial value: 0
Total protons pumped when the reducing agent is NADH.

Biology Open • Supplementary information

P_v
Implementation Name: `P_v`
Units: mmHg
Initial value: $P_{v,n}$
Venous blood pressure.

Pa_{CO_2}
Implementation Name: `Pa_CO2`
Units: mmHg
Initial value: $Pa_{CO_{2,n}}$
Arterial partial pressure of carbon dioxide.

S_{a,O_2}
Implementation Name: `SaO2sup`
Units: dimensionless
Initial value: $S_{a,O_2,n}$
Arterial oxygen saturation.

$S_{c,O_2,n}$
Implementation Name: `ScO2_n`
Units: dimensionless
Initial value: $\dfrac{S_{a,O_2,n} + S_{v,O_2,n}}{2}$
Normal capillary oxygen saturation.

$\sigma_{e,n}$
Implementation Name: `sigma_en`
Units: mm Hg
Initial value: $\sigma_{e,0}\left(\exp\left(\dfrac{K_\sigma\,(r_n - r_0)}{r_0}\right) - 1\right) - \sigma_{coll}$
Normal elastic stress in blood vessel walls.

$S_{v,O_2,n}$
Implementation Name: `SvO2_n`
Units: dimensionless
Initial value: $\dfrac{HbO_{2,v,n}}{Hb_{tot,n}}$
Normal venous oxygen saturation.

$T_{e,n}$
Implementation Name: `T_en`
Units: mm Hg cm
Initial value: $\sigma_{e,n}\,h_n$
Normal elastic tension in the blood vessel walls.

$T_{max,0}$
Implementation Name: `T_max0`
Units: mm Hg cm
Initial value: $\dfrac{T_{max,n}}{1 + k_{aut}\,\mu_n}$
Maximal muscular tension under normal regulatory stimulus ($\mu = \mu_n$).

$T_{max,n}$
Implementation Name: `T_maxn`
Units: mm Hg cm
Initial value: $\dfrac{T_{m,n}}{\exp\left(-\left|\frac{r_n - r_m}{r_t - r_m}\right|^{n_m}\right)}$
Normal maximal muscular tension.

$$T_{m,n} = (P_{1,n} - P_{icn})\,r_n - T_{e,n}$$

Biology Open • Supplementary information

Implementation Name: T_mn
Units: mm Hg cm
Initial value: 0
Normal muscular tension in the blood vessel walls.

$v_{CO_2,2,n} = Pa_{CO_{2,n}}$
Implementation Name: v_c2n
Units: mmHg
Initial value: 0
Normal filtered CO_2 partial pressure affecting metabolism.

$v_{CO_2,n} = Pa_{CO_{2,n}}$
Implementation Name: v_cn
Units: mmHg
Initial value: 0
Normal filtered carbon dioxide partial pressure.

$v_{O_2,n}$
Implementation Name: v_on
Units: mM
Initial value: $O_{2,c,n}$
Normal filtered capillary oxygen concentration.

$v_{P_a,n} = P_{a,n}$
Implementation Name: v_pn
Units: mmHg
Initial value: 0
Normal filtered arterial blood pressure.

$v_{u,n}$
Implementation Name: v_un
Units: dimensionless
Initial value: u_n
Normal filtered demand.

$$V_{a,n} = \frac{V_{tot,n}}{1 + VArat_n}$$
Implementation Name: Vol_artn
Units: dimensionless
Initial value: 0
Normal relative arterial blood volume.

$$V_v = \frac{V_{tot,n}\, VArat_n}{1 + VArat_n}$$
Implementation Name: Vol_ven
Units: dimensionless
Initial value: 0
Relative venous blood volume.

$HbO_{2,a} = Hb_{tot}\, S_{a,O_2}$
Implementation Name: XOa
Units: mM
Initial value: $HbO_{2,a,n}$
Arterial concentration of oxygen bound to haemoglobin.

$HbO_{2,a,n}$
Implementation Name: XOa_n
Units: mM
Initial value: $Hb_{tot,n}\, S_{a,O_2,n}$
Normal arterial concentration of oxygen bound to haemoglobin.

$$HbO_{2,v,n} = \frac{CBF_n\, HbO_{2,a,n} - J_{O_{2,n}}}{CBF_n}$$
Implementation Name: XOv_n
Units: mM
Initial value: 0
Normal venous concentration of oxygen bound to haemoglobin.

Biology Open • Supplementary information
